# A low-cost automized anaerobic chamber for long-term growth experiments and sample handling

**DOI:** 10.1016/j.ohx.2021.e00237

**Published:** 2021-10-12

**Authors:** Achim J. Herrmann, Michelle M. Gehringer

**Affiliations:** Department of Microbiology, Technische Universität Kaiserslautern, Paul Erlich Strasse, 67665 Kaiserslautern, Germany

**Keywords:** Anaerobic workstation, Glove box, Anaerobic, Anoxic, Culturing system

## Abstract

The handling of oxygen sensitive samples and growth of obligate anaerobic organisms requires the stringent exclusion of oxygen, which is omnipresent in our normal atmospheric environment. Anaerobic workstations (aka. Glove boxes) enable the handling of oxygen sensitive samples during complex procedures, or the long-term incubation of anaerobic organisms. Depending on the application requirements, commercial workstations can cost up to 60.000 €. Here we present the complete build instructions for a highly adaptive, Arduino based, anaerobic workstation for microbial cultivation and sample handling, with features normally found only in high cost commercial solutions. This build can automatically regulate humidity, H_2_ levels (as oxygen reductant), log the environmental data and purge the airlock. It is built as compact as possible to allow it to fit into regular growth chambers for full environmental control. In our experiments, oxygen levels during the continuous growth of oxygen producing cyanobacteria, stayed under 0.03 % for 21 days without needing user intervention. The modular Arduino controller allows for the easy incorporation of additional regulation parameters, such as CO_2_ concentration or air pressure. This paper provides researchers with a low cost, entry level workstation for anaerobic sample handling with the flexibility to match their specific experimental needs.

## Graphical abstract

**Figure:**
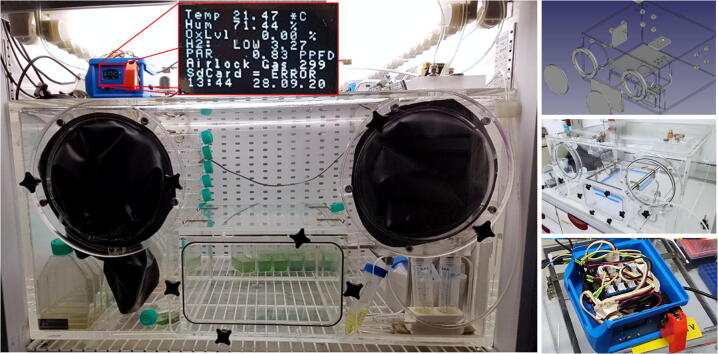

**Specifications table** [please fill in right-hand column of the table below].

**Table:** 

Hardware name	*Anaerobic chamber*
Subject area	• Biological Sciences (e.g. Microbiology and Biochemistry) • General
Hardware type	• Biological sample handling and preparation
Open Source License	MIT License
Cost of Hardware	∼2000 €
Source File Repository	10.17605/OSF.IO/Y2G8H

## Introduction hardware in context

Handling oxygen sensitive samples can provide a challenge in modern earth’s atmosphere, as oxygen is the second most abundant gas and even trace amounts can lead to the rapid and irreversible oxidation of a sample. Handling oxygen sensitive samples can be further complicated if the sample is alive, like obligate anaerobic organisms, where oxygen and its radical forms acts as a potent toxin, oxidizing important proteins and catalytic metal ligands [2]. Cheap solutions like sealed glass tubes or flasks with rubber lids used in combination with syringes, produce a lot of waste and their use is limited if complex sample handling is required. This hardware offers flexibility in manual sample processing, in an anaerobic environment, in an affordable, programmable anaerobic workstation.

## Hardware description

This paper describes the construction (Table 1) of a low-cost alternative to commercial anaerobic chamber with advanced features usually only found in high cost, automated anaerobic chambers. Barebone anaerobic workstations with glove ports can easily cost more than 10.000 €, even without “comfort” functions like an automated airlock or automatic maintenance of an anaerobic atmosphere. Advanced features like humidity or CO_2_ control quickly add 5,000 to 10,000 € to the cost, depending on the feature and company in question. Here we describe the complete build instructions to a cheap, Arduino based, automated anaerobic workstation, which can be used in the cultivation of bacteria or for general sample handling. In contrast to other DIY solutions, the automated control allows for long term experiments or storage of samples without user input. Additional features like CO_2_ regulation or pressure control can be added as needed per experimental requirement, even after the final installation.

**Table 1:** Numbered part list with the needed amounts.

Part Nr.	Part Name	Amount
1	Mainbody	1
2	FrontDoor	1
3	GloveGasket	2
4	GloveLid	2
5	AirlockTop	1
6	GasPortAirlock	1
7	GasPortTop	2
8	FanHolder	12
9	HolderSensor	1
10	LSensorHolder	1
11	ScrewBacking25	9
12	ScrewBackingFlat	7
13	ChamberPipeInlet	1
14	ChamberPipeOutlet	1
15	OverpressurePipe	1

Advantages of this design:

automatic regulation of humidity and H_2_ (as oxygen reductant) concentration
Logger for monitoring environmental conditions during the experiments
Automatic air-lock purging
Easily expandable for individual requirements like controlling the CO_2_ concentration
Compact design allows for the integration into growth chambers/cell incubators and/or minimal impact on available lab space

Possible use cases:

Growth of anaerobic organisms
Growth of oxygen producers like cyanobacteria under anaerobic conditions
Handling and storage of oxygen sensitive samples
Radioactive labeling experiments under anaerobic conditions

## Design files

Design Files Summary

**Table:** 

Design file name	File type	Open source license	Location of the file
Mainbody	CAD .fcstd	MIT	10.17605/OSF.IO/Y2G8H
FrontDoor	CAD .fcstd	MIT	
GloveGasket	CAD .fcstd	MIT	
GloveLid	CAD .fcstd	MIT	
AirlockTop	CAD .fcstd	MIT	
GasPortAirlock	CAD .fcstd	MIT	
GasPortTop	CAD .fcstd	MIT	
FanHolder	CAD .fcstd	MIT	
HolderSensor	CAD .fcstd	MIT	
LSensorHolder	CAD .fcstd	MIT	
ScrewBacking25	CAD .fcstd	MIT	
ScrewBackingFlat	CAD .fcstd	MIT	
ChamberPipeInlet	CAD .fcstd	MIT	
ChamberPipeOutlet	CAD .fcstd	MIT	
OverpressurePipe	CAD .fcstd	MIT	
CompleteAssembly	CAD .fcstd	MIT	
ArduinoCad	Electronic .dsn	MIT	
SS3_eng	Software .ino	MIT	
SS3_eng_setup	Software .ino	MIT	

### File description

**Fig. 1:**
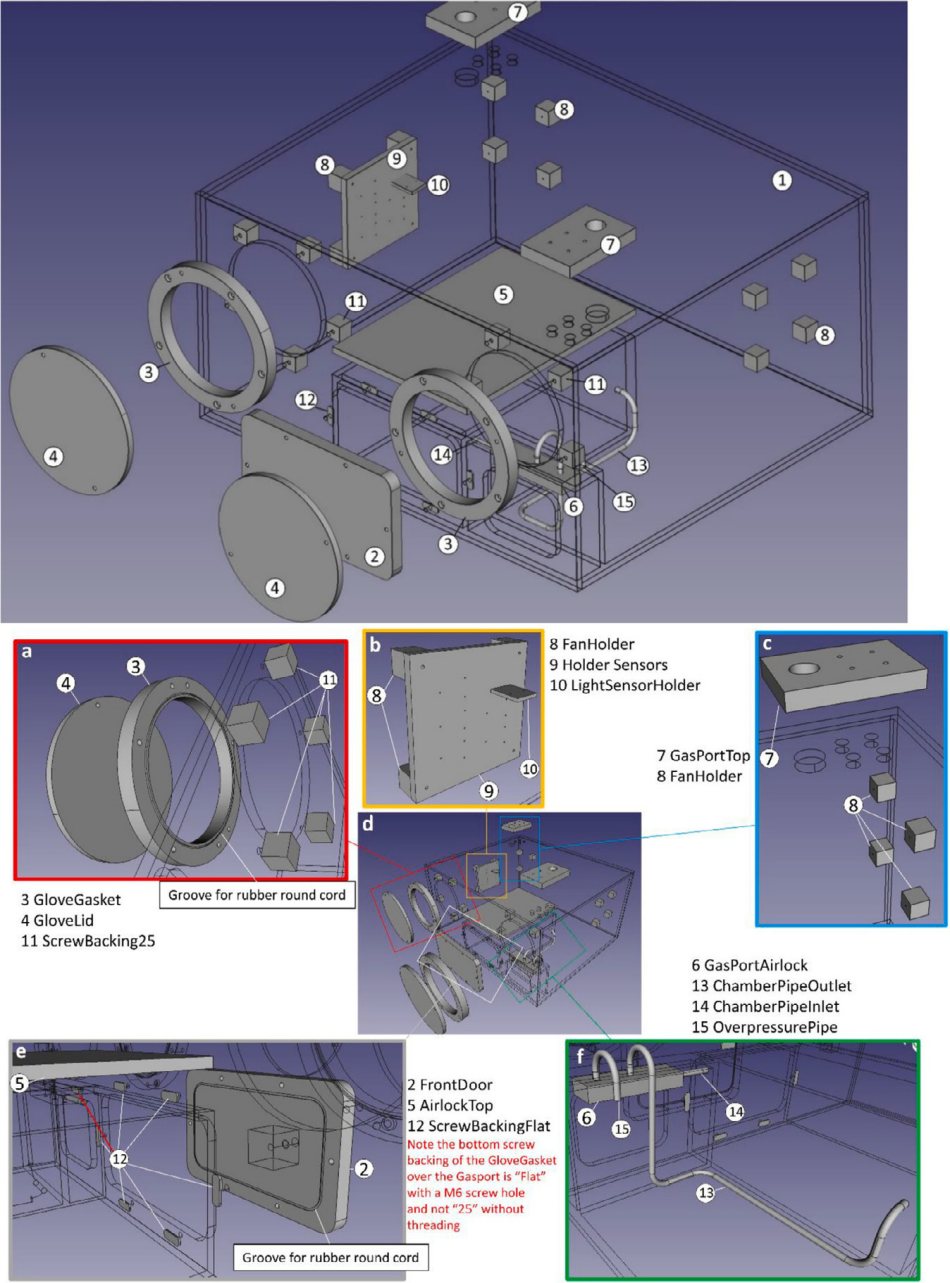
Schematic overview of all the PMMA parts needed for the assembly of the anaerobic station (top) with detailed views (bottom). Panels illustrate the following: (a) Glove port assembly, (b) Sensor assembly, (c) Gas port, (d) Overview, (e) Airlock doors and (f) Gas piping. Parts are numbered according to the list in Table 1.

The files named “Mainbody” through to “ChamberPipeOutlet” describe the parts needed in the assembly of the PMMA (Poly(metyl-2-methylpropenoate)/Perspex) body of the anaerobic workstation and are shown assembled in the file “CompleteAssembly” and Fig. 1 top All CAD files were created using FreeCAD 0.18, build 4.

“Mainbody” describes the body onto which all parts are assembled. It was assembled from multiple slabs of PMMA, which were cut and drilled, before being glued together. In order to reduce costs, the bottom, sides and back plate could be made from a cheaper material, as only the front and top part needs to be translucent in order to see inside.

“FrontDoor” describes the front door of the airlock, which seals the box hermetically. The groove on the inside should be fitted with the round cord to ensure an airtight seal. The front door is mounted to the main body with the 6 M6 threaded rods (40 mm) with star handles.

“GloveGasket” describes the gasket to secure the gloves in place during operation and ensure an airtight seal. The groove on the inside should be fitted with the a rubber round cord to ensure an airtight seal. For an easier installation of the gloves inside the gaskets, clear sticky tape can be used to hold the gloves in place during instalation. The gasket are mounted with the M6 head cap screws. Note that the screw hole directly in front of the gas port is not backed by a 25 mm screw backing and the shorter M6x22 screw must be used.

“GloveLid” describes a cover for the glove holes, if the workstation is not in use, to prevent the gloves from sticking out due to the internal over pressure. It can be fixed to the GloveGasket with the 4 M6 threaded rods (35 mm) with star handles while the gloves are not used.

“AirlockTop” describes the top cover of the airlock. Holes for the toggle fastener are not described, as they are part dependent, and must be adjusted for a tight fit with the seal strip. This must be done before the mainbody is sealed as the cover is too big to fit through the airlock.

“GasPortAirlock” describes a block with screw fittings for the airlock gas in- and outlet, as well as the main chamber gas outlet. It needs to be glued in the Gas outlet bay (Fig. 6).

“GasPortTop” describes a block with screw fittings for the gas inlets and cable port. The biggest hole is for a Rubber stopper diaphragm, if the drawing of gas with a syringe is needed. They need to be glued over the corresponding access holes of the mainbody.

“FanHolder” describes single blocks for attachment of the pc fans and the sensor holder. These need to be glued in sets of four, with the required spacing inside the main body, to ensure a constant air flow for a uniform removal of oxygen.

“HolderSensor” describes the mounting plate for up to five Grove sensor shields and the “LSensorHolder” for an upright mounting of the light sensor. Needs to be mounted on one set of “FanHolder” with 4 M3x35 screws.

“ScrewBacking25/-Flat” describe pieces of PMMA which cover the screw holes in the main body for an airtight compartment and need to be glued above the indicated screw holes in the main body.

“ChamberPipeInlet/-Outlet” describe the pipes needed to connect the airlock in and outlet to the “GasPortAirlock”, and need to be glued in the corresponding holes.

“OverpressurePipe” describes the pipe connected to the “GasPortAirlock”, where pressure from the main box is vented. It is needed to prevent the gloves from accidentally obstructing the gas outlet and is glued into the “GasPortAirlock”.

“CompleteAssembly” shows the complete assembly of all the parts so far described (Fig. 1 top).

“ArduinoCad” shows the layout of the electric connections of the sensors to the Arduino controller, as well as the power supply and relay cabling. It was created using TinyCad version 3.00.02.

“SS3_eng” is the main software running on the Arduino controller during operation of the anaerobic workstation.

“SS3_eng_setup” is used during the initial sensor and timing setup and for verification of sensors functionality (see 6.2).

## Bill of materials

The bill of materials is uploaded to the open science framework at https://doi.org/10.17605/OSF.IO/Y2G8H .

## Build instructions

### Assembly of PMMA Mainbody

Assemble the PMMA body of the anaerobic box in Fig. 1 as per construction file (“CompleteAssembly”). Before completing the “Mainbody” by gluing the “Mainbody” lid in place be sure to install the following internal parts as a later installation might be difficult or impossible:

1. Cover all the screw holes/ports for the attachment of the “FrontDoor” and the “GloveGasket”s with “ScrewBacking25/-Flat” parts for an airtight seal. The 6 threaded holes of the “FrontDoor” and the single threaded hole in the Airlock Port need to be covered with “ScrewBackingFlat” (Fig. 1e). The remaining 9 holes of the “GloveGasket”s need to sealed with “ScrewBacking25” (Fig. 1a).
2. Cover the top of the Airlock body walls with the sponge rubber seal strip. Attach the toggle fastener hinges to the “AirlockTop” part and place it on the Airlock body (Fig. 2c). The boreholes for the toggle fasteners are not specified in the blueprints and should be adjusted to ensure a tight fit with the underlying sealing strip if the toggle fasteners are engaged. Mark the position were the toggle fastener clamps need to be placed on the Airlock and then permanently attach them with M3 Screws and nuts (Fig. 2c). The bore holes were additionally sealed with glue for an airtight seal. Note that the “AirlockTop” part must be placed inside the “Mainbody” before gluing the lid of the “Mainbody” into place, as it is too large to be fitted in through the airlock afterwards.

**Fig. 2:**
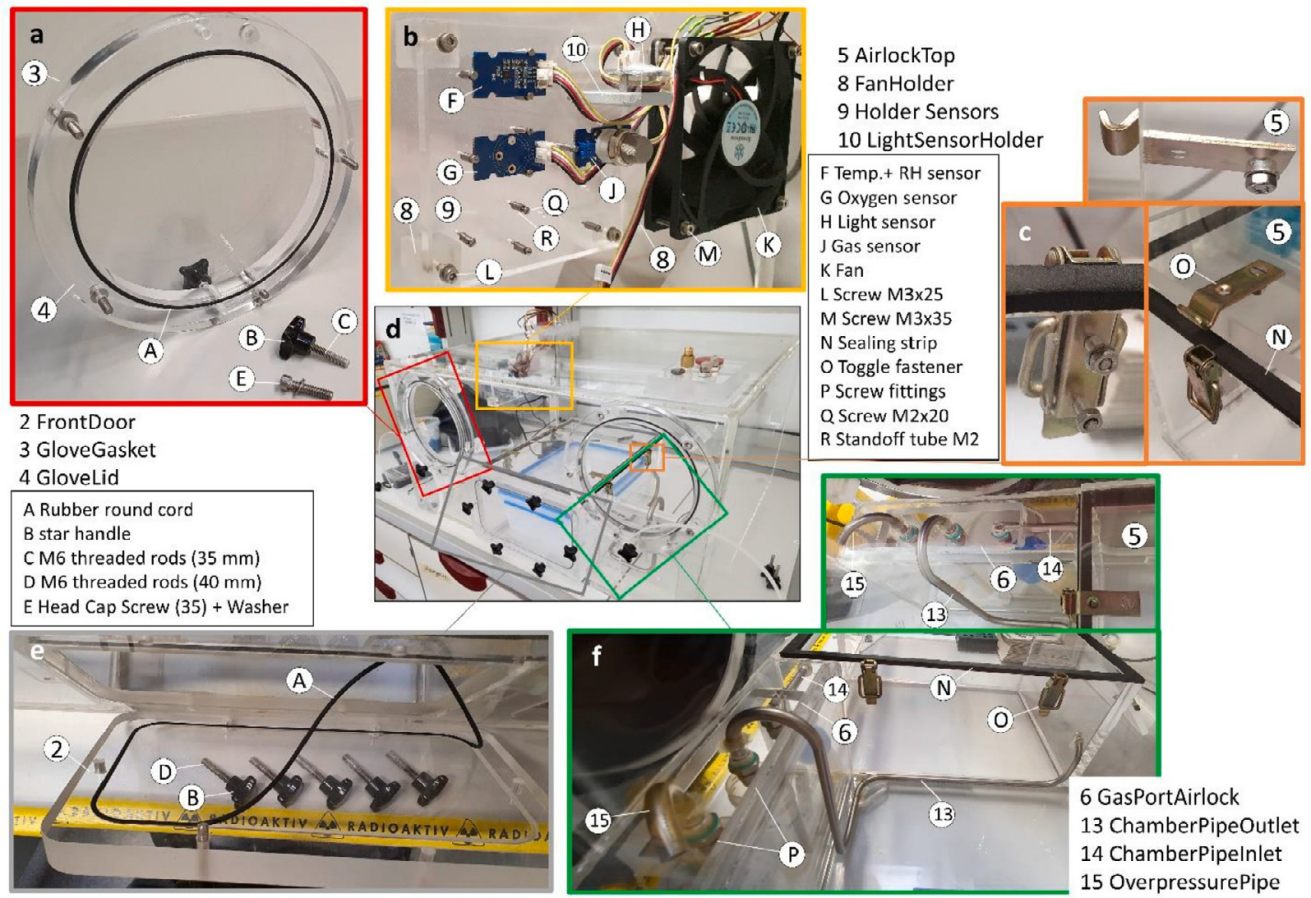
A: Completely assembled PMMA body of the anaerobic chamber with accessories, central panel (d). Shown in details are : (a) Complete glove port assembly, (b) Sensor assembly without oxygen sensor, (c) Toggle fastener on the airlock, (e): Airlock front door assembly and (f) Gas piping.
3. All “FanHolder” parts should be glued into place. For the correct spacing this can be done with the fans or “HolderSensor” already attached. The sensors should not be mounted during this step, as fumes from the glue could damage the gas sensors (Fig. 1b + c, Fig. 2b). Note: The left fan was glued to the left wall instead of onto the back, as indicated in the blueprints.
4. The “GasPortAirlock” should be glued into the indicated place inside the Airlock port. The piping (“ChamberPipeInlet/-Outlet” and “OverpressurePipe”) was then attached into the corresponding holes and glued into place (Fig. 1f, Fig. 2f).

After the installation of all this parts the lid of the “Mainbody” can be glued into place and the 2 “GasPortTop” parts can be attached over the corresponding holes on the lid (Fig. 1c).

Also the rubber round cord needs to be cut, rejoined and fitted inside the groves of the “GloveGasket” and the “FrontDoor” for an airtight enclosure (Fig. 1a + e, Fig. 2a + e).

### Installation of accessories and electronics

**Fig. 3:**
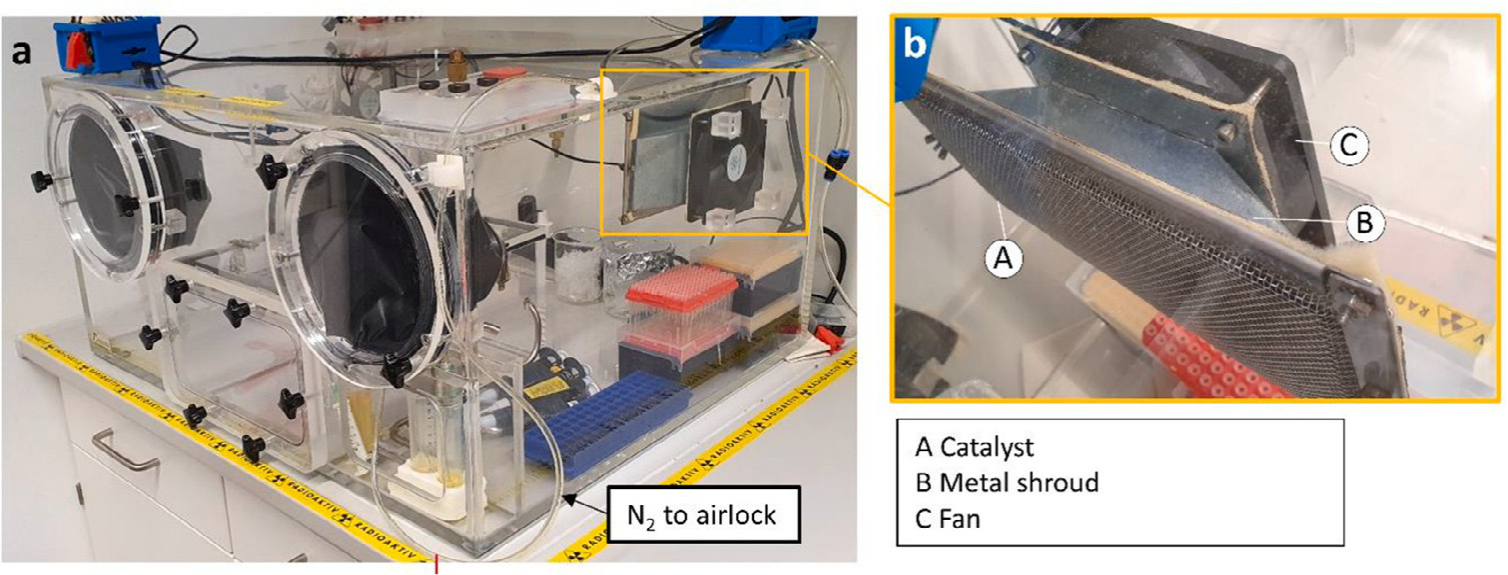
Completely assembled PMMA body of the anaerobic chamber with accessory and electronic (a). Shown in detail is the complete catalyst assembly with metal shroud and fan (b).

After the complete assembly of all the PMMA parts and curing of the glue the electronics and the catalyst can be installed. Mount the fans on the fan holders and attach the catalyst (StakPak) in front of one of the fans (Fig. 3b). In our case, this was achieved by a folded metal shroud made of 2 mm steel (Fig. 3b -B). As a cost saving measure, a catalyst can be easily made from scratch: 10 to 20 g of Palladium on Carbon (5%) pellets (2–3 mm) should be encased between two layers of fine stainless-steel wire mesh. The sides can then be sealed by folding them and stapling them shut. Do not use flammable substances in the construction as the catalyst can become hot during operation and could melt plastic or char cardboard.

**Fig. 4:**
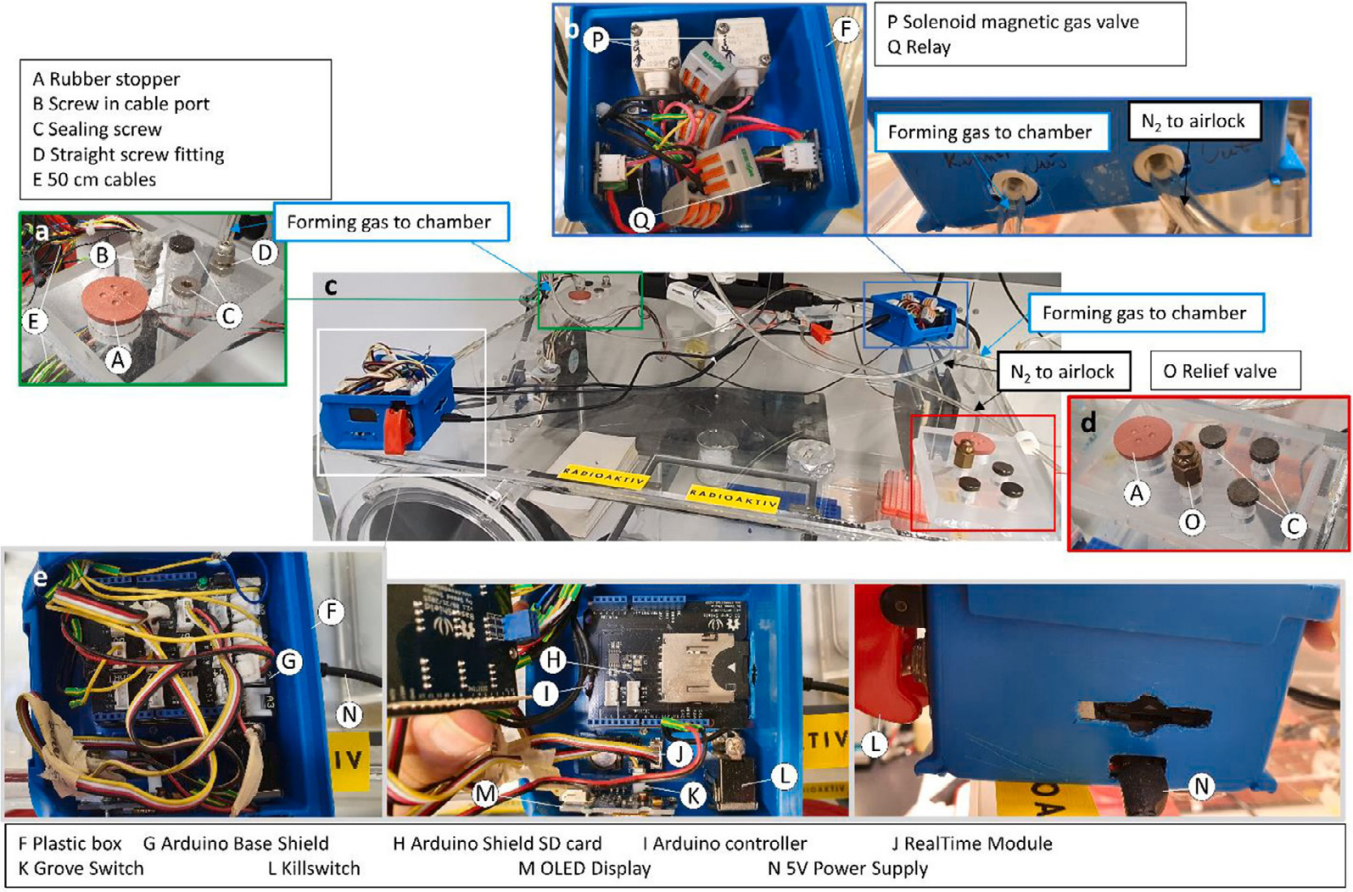
Completely assembled PMMA body of the anaerobic chamber top with accessory and electronic (c). Shown are in details: a: Upper left gas port. b: Gas regulation unit with relays and valves. d: Lower right gas port. e: Arduino control unit. Note the special separation of the Arduino control and gas regulation unit to reduce interference during operation of the magnetic valves.

**Fig. 5:**
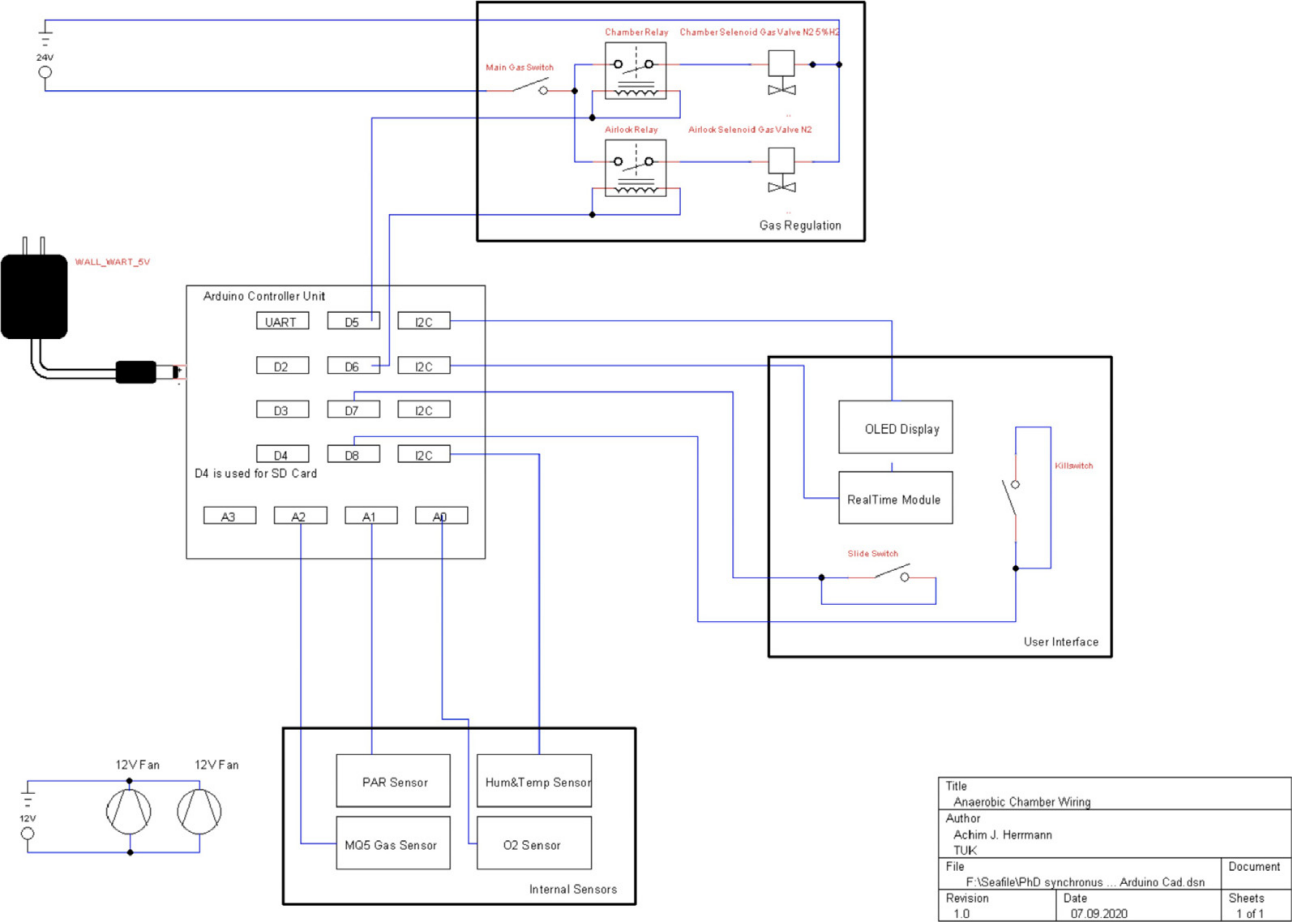
Cabling diagram of the electronic components of the anaerobic box.

Fan power and connection cables are passed through a screw-in cable port and the port sealed airtight with either adhesive putty or epoxy resin (Fig. 4a). Connect all sensor cables to the Arduino (Fig. 4e) as seen in Fig. 5 or in the “Arduino CAD” file (TinyCAD v3.00).

**Fig. 6:**
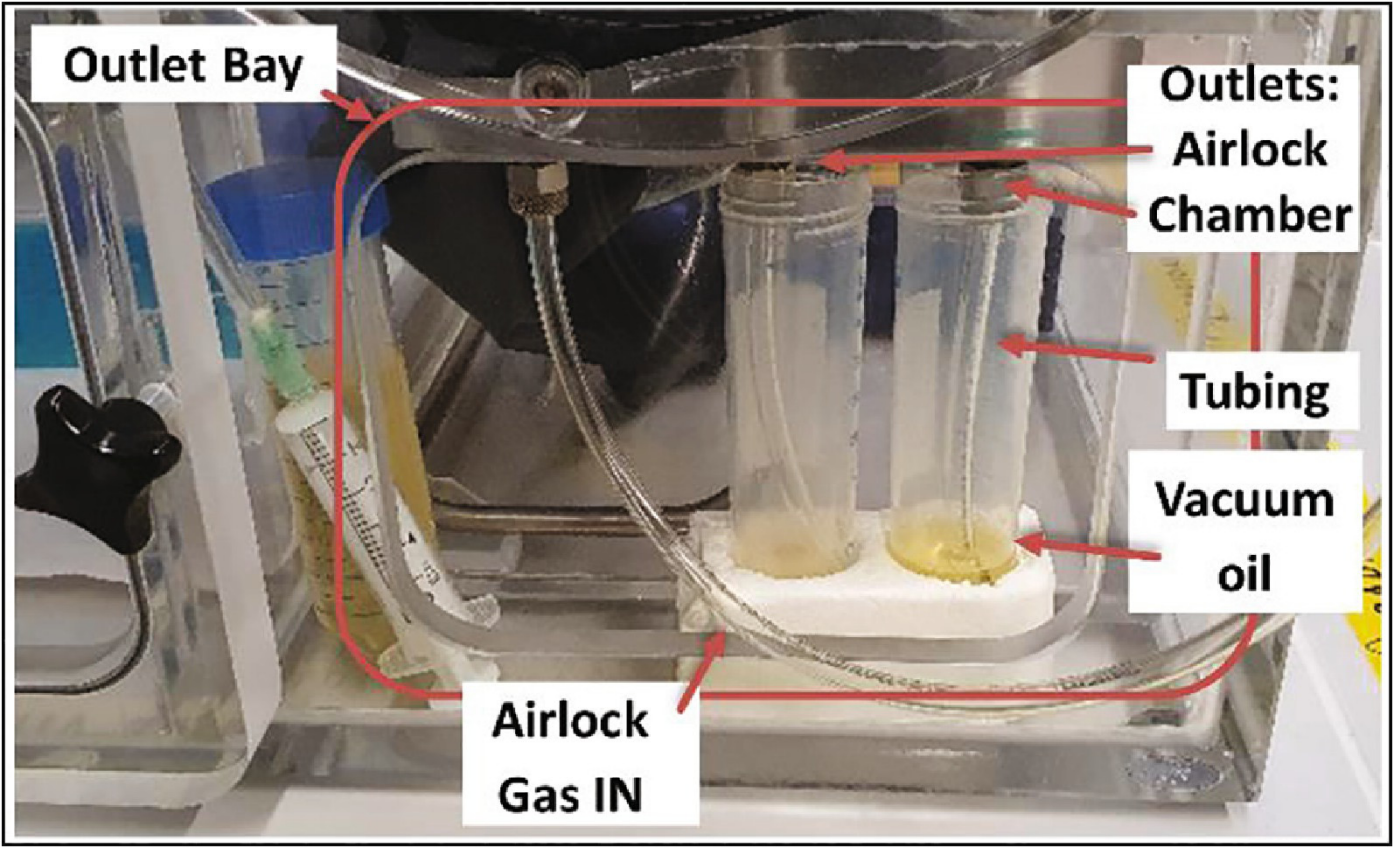
Gas outlet bay with 50 ml test tubes filled with vacuum oil filed just above the tube opening.

**Fig. 7:**
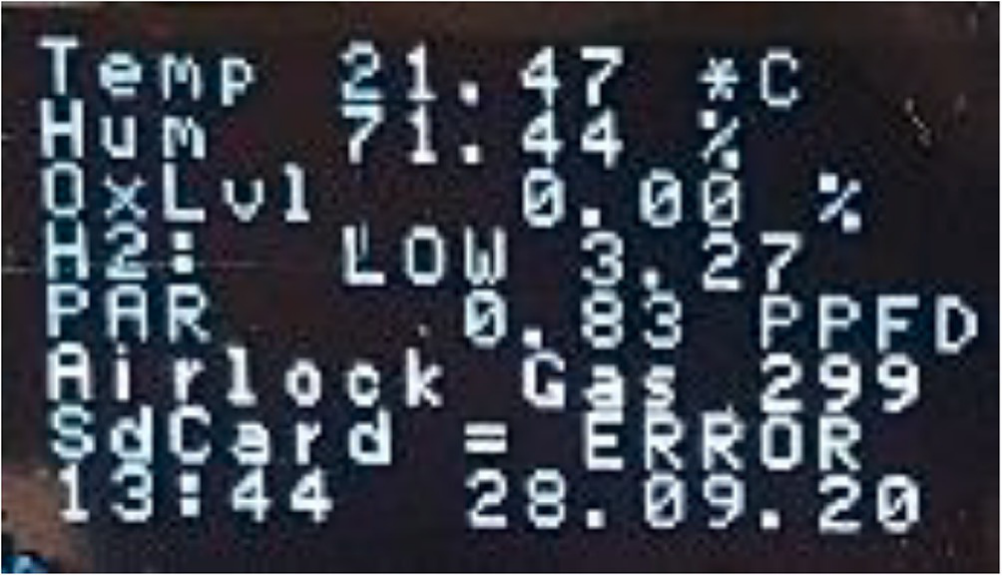
User interface on the OLED display.

The Arduino controller must be electronically shielded or spatially separated from the magnetic gas valves and their power source, as their operation can create strong magnetic fields and hence system instability. In our case, this was achieved by placing the Arduino in one case made from a plastic storage box, while the gas valves with their relays were placed in a second plastic box and connected with 40 cm of wiring (Fig. 4c).

The sensors used in this configuration of the anaerobic chamber are sensors for reducing gases, oxygen, humidity, temperature and light. Their cabling is shown in Fig. 5 and they are installed on the “HolderSensor” with “L(ight)SensorHolder” (Fig. 1,Fig. 2b)

**Fig. 8:**
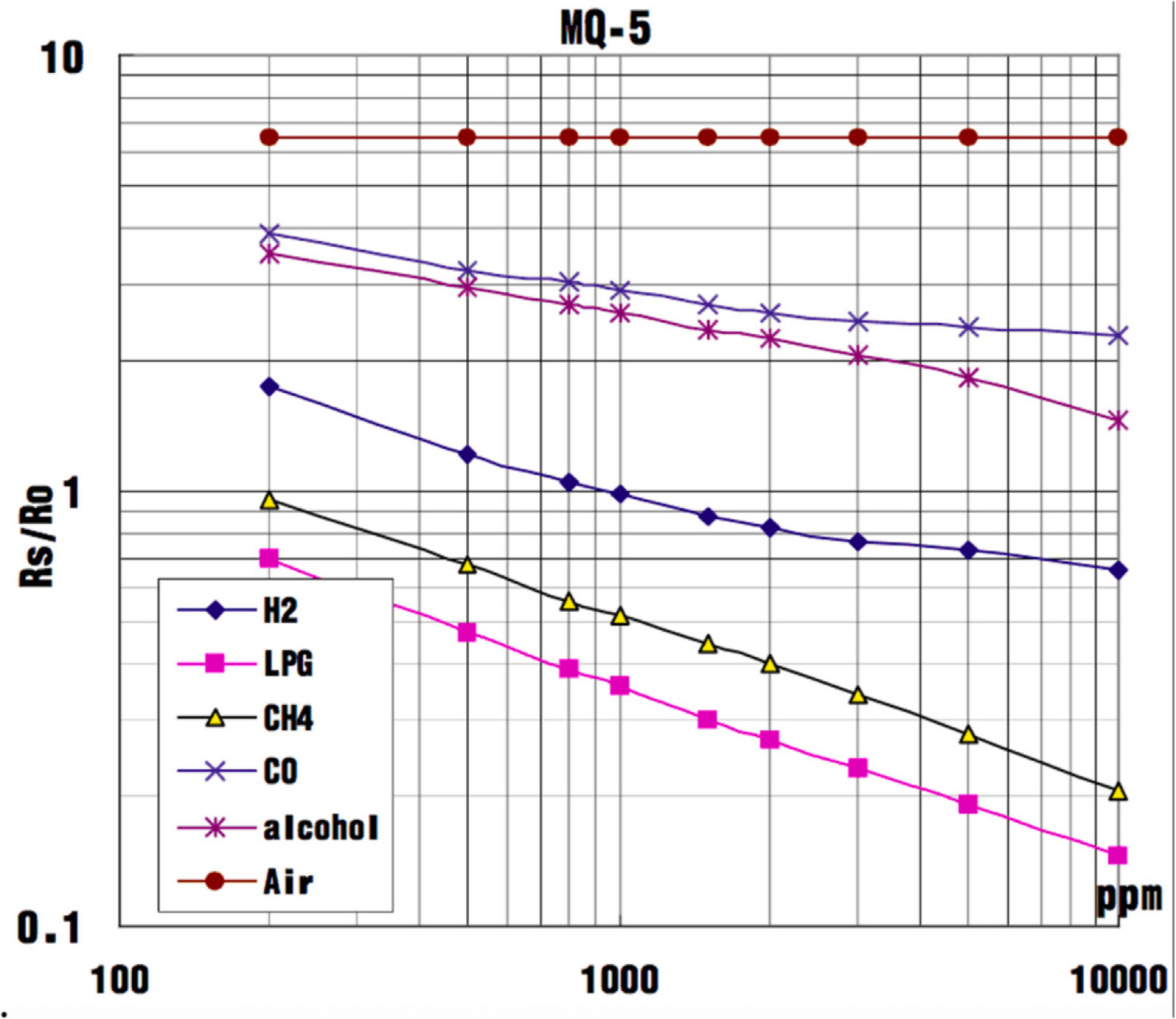
R_S_ (Reading Sensor)/ R_0_ (Reading Zero) value gives an indication of the gas levels in the atmosphere. Note that the sensor will react to other gases in the atmosphere, which should be taken into account during the operation of the automated gassing system (https://wiki.seeedstudio.com/Grove-Gas_Sensor-MQ5/; 13.10.2020).

The reducing gas sensors (MQ-5) is used as an indicator for the presence of H_2_ in the box atmosphere, an important parameter as H_2_ is consumed in the process of oxygen removal by the palladium catalyst. This sensor detects the presence of reducing gases in a thin, heated tin dioxide layer, which changes resistance according to the redox state of the surrounding atmosphere and can be used to estimate the concentration of different gases (see Fig. 8). To measure the change in resistance, the resting resistance needs to be calibrated using the Arduino Program “SS3_eng_setup”. Connect the microcontroller to the MQ-5 as described in Fig. 5 and open the serial monitor. Note the R_0_ value of the H_2_ sensor in ambient air. This value needs to be adjusted in the main program “SS3_eng”. Additionally, the sensor functionality can be verified by exposing it to reducing gases or vapors like ethanol or isopropanol vapour.

After measuring the R_0_ value, adjust the lines in the main program “SS3_eng”:

## //H2 SetUp

**const float R0 = 6.5; // resting voltage of H2 Sensors; Determine by using “H2 setup”**

The oxygen concentration in the box’s atmosphere is measured using an electrochemical oxygen sensor (ME2-O2, Range 0–25% O_2_, and Accuracy ± 0.05%). The sensor measures the current, which is produced during the reaction of oxygen with its electrode material, similar to a zinc-oxygen battery. As the inflow of oxygen is limited by a small capillary, the current is proportional to the concentration of oxygen in the surrounding atmosphere. As the electrode is used up in the reaction with oxygen, the sensor should be stored in its airtight capsule (preferably made anoxic) if the anaerobic box is not in use for a prolonged time (Note the missing sensor in Fig. 2b).

The setup file also sets the Real Time Chip. Adjust the files in void setup in SS3_eng_setup” according to your current date and time:

## The above process sets the time to the specified values every time the code is run and should be deleted after the initial setup or the displayed time may be inaccurate.**void setup() {**

**…;**

**clock.begin(); // fill in the correct date and time to initialize clock [3]**

**clock.fillByYMD(2017,1,19); //Jan 19,2017**

**clock.fillByHMS(15,28,30); //15:28 30″**

**clock.fillDayOfWeek(SAT); //Saturday, arguments [MON, TUE, WED, THU, FRI, SAT, SUN]**

**clock.setTime(); //write time to the RTC chip**

**}**

The temperature and humidity are measured by a combined sensor (SHT31, Range 0–100% RH and −40 to 125 °C, Accuracy ± 2% RH and ± 0.3 °C) and are determined by the change in resistance of the sensors depending on the environmental factors.

The light sensor (APDS-9002) uses a light photosensor to indicate presence of light (Range 0 – 1000 Lux, Accuracy ± 2% of measured value), which can be useful if the box is used for the cultivation photosynthetic organisms. The sensor can be calibrated with a PAR meter in order to estimate the light intensity inside the box. If the desired light intensity is outside the sensor’s detection range, the sensor can either be used with semitransparent folies to reduce the light intensity or as a simple on/off indicator.

Further information on the function, principal and application of the utilized sensors can be found on: https://wiki.seeedstudio.com

Safety instructions:

The anaerobic workstation uses non-toxic, asphyxiating gases and should not be operated in confined spaces without ventilation. If forming gas with ≤ 5% H_2_ is used, there is no explosive risk, as the minimal flammability limit cannot be reached by mixing forming gas with ambient air. The risk of electric shock is minimal as the maximal voltage used is only 24 V.

## Operation instructions

The ends of the chamber/airlock gas outlet tubes must be submerged in water or oil to prevent the inflow of ambient air. This can be achieved either by placing the end of a short section of stiff tubing inside a liquid filled reaction tube or similarly sized vessel inside the outlet bay or running the tubing to an external gas washing flask or similar device. Care should be taken to not completely fill the vessels, as the immersion depth of the tubing correlates to the maximum internal overpressure during gassing operations. High internal pressure can make the gloves rather stiff and unpleasant to handle while also posing the risk of an outward spillage of oil/water during a rapid insertion of the gloves. Likewise, a rapid retraction of the gloves poses the risk of sucking in liquid and ambient air contaminating the internal atmosphere/ surroundings. Therefore, care should be taken while inserting and removing one’s arms and, if necessary, gas can be manually inserted by activating the manual gas injection switch to alleviate an eventual negative pressure. Tip: If the liquid level in the container, which houses the outlet from the airlock, is lower than in the chamber outlet, less ambient air will enter the main chamber during an airlock cycle.

N_2_ can be used in certain steps as a cost saving measure. All steps describing the usage of N_2_ gas can also be performed with forming gas if no N_2_ is available.

### Initial setup

1. Make sure all tubes are connected, all unused screw ports are covered by sealing screws and all internal sensors are working. Fill up the outlet vessels with oil/water to slightly cover the tube endings (∼5 mm).
2. All necessary equipment like pipettes, test tube holders e.g. should be inserted before making the chamber anaerobic, as this can save time otherwise spent on unnecessary airlock cycles.
3. (Optional) Flush the box with N_2_ gas for 20 min to bring down the O_2_ levels and save forming gas.
4. Switch on the controller with the connected forming gas. Forming gas injection will start to remove the remaining O_2_. Humidity should rise as O_2_ reacts with H_2_ to form H_2_O. This exothermic reaction can heat up the catalyst and damage delicate equipment if they are in direct contact.
5. The controller should auto adjust the injections of forming gas and N_2_ until an atmosphere is established within the parameters set for humidity and enough H_2_ gas to remove all O_2_ present in the anaerobic chamber. As the frequency of gas injections during setup is most likely over the safe operating threshold during normal operation, the gas override switch should be switched to ON (7.2).

### User interface

The OLED display (Fig. 7) shows all relevant environmental conditions during operation:

1. Temperature in °C
2. Relative Humidity in % saturation at the temperature in line 1
3. Oxygen levels in % with an error of 0.03%
4. Indication of the H_2_ level in the chamber with:
  a. NONE ∼ H2 < 1%
  b. LOW ∼ H2 < 3%
  c. NORM ∼ H2 greater than 3%

The number is displays the ratio of the Rs (Reading Sensor) over R_0_ (Reading Zero) ratio, NOT H_2_ in % (Fig. 8) and is a rough indication of the presence of hydrogen in the atmosphere. As the sensor can also detect alcohols, alcohol-based disinfectants should not be used inside of the glove box, otherwise the auto injection of forming gas will not work.

5. PAR (Photosynthetic Active Radiation) in PPFD (Photosynthetic Proton Flux Density). Only works if the light sensor is calibrated, otherwise just an indicator of the current day-night / light–dark cycle
6. Status of the automatic gas injection
  a. GASSING ERROR: The auto injection was disabled because gas was injected too often in a short interval, hinting at a leak or sensor malfunction. To reset this message and enable auto injection flip the OVERRIDE switch to ON and return to OFF.
  b. GAS OVERRIDE: Displays the status of the OVERRIDE switch as ON.
  c. AIRLOCK GAS = n: Automatic gassing of the airlock with n seconds remaining
7. Status of the SDCard on which all data is automatically saved
  a. ERROR = SD Card is not mounted, either no SD Card is inserted or it could not be mounted
  b. OK = SD Card is mounted and data gets saved every minute
8. Time and date

**Fig. 9:**
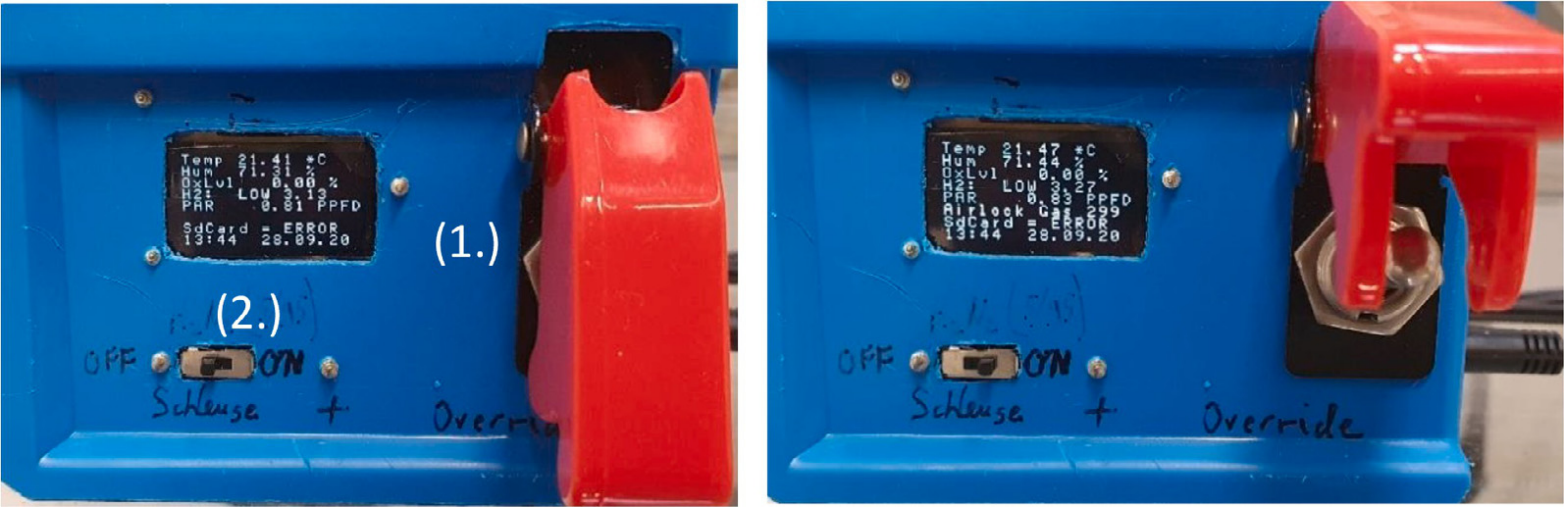
Operator control interface with the Killswitch (1) and Slide Switch (2) disengaged (left) and engaged (right). Note the change in the OLED display line 6.

The controller has two operation modes, which can be controlled by switching the Killswitch (Fig. 9, 1) and Sliding Switch (Fig. 9, 2) to the corresponding positions.

1. Killswitch (1) is OFF:
a. Slide Switch (2) is OFF:
  Emergency Stop Function is active: the controller checks if 5 consecutive gas injections were triggered within 10 s of each other (indication of a leakage or an empty gas bottle) and stops further gas injection without user input.
  i. Slide Switch (2) is ON:

Manually injects forming gas until switched back to OFF. Useful to prevent negative pressure while operating the gloves.

2. Killswitch (1) is ON:
a. Slide Switch (2) is OFF:
Display shows GAS OVERRIDE. Resets the emergency Stop Function and disables it while the Killswitch (1) is engaged. Useful for the first start-up of the box, where the rapid injection of forming gas would trigger the emergency stop function (1.a).
b. Slide Switch (2) is ON:

Floods the airlock chamber with N_2_ for 300 s and displays the remaining time on the display. The duration can be adjusted in the program file in the line “const int GasFlowSchleuseDuration = 300; // How long should the air lock be gased (s)” Gassing can be stopped by disengaging any off the input switches (1 or 2) to OFF. In order to minimize the inflow of ambient air from the airlock chamber into the main body, a tight fit of the inner airlock top cover must be ensured. During the first minute of gassing, the airlock front door can also be slightly opened to minimize the infiltration of oxygen rich gas into the mainbody of the anaerobic chamber.

### Cleaning and maintenance

Avoid using solvents during the cleaning and disinfection of the anaerobic chamber as the MQ5 sensor detects not only H_2_ but also hydrogen in hydrocarbons. It may give a false indication of H_2_ levels, if volatile solvents like ethanol or isopropanol are present in the anaerobic chambers atmosphere. Solvents can also lead to cracking or cloudiness of the PMMA material and adsorb onto the activated charcoal catalyst, reducing its activity. In order to remove adsorbed solvents from the catalyst, it can be baked overnight at 200 °C (Caution: depending on the quantities adsorbed on the catalyst this can pose an explosion hazard!!!). Therefore, only use water and mild detergents during cleaning operations if possible.

Care should be taken during the incubation of organisms that produce or require SO_2_/H_2_S or CO as these gases can lead to a poisoning of the palladium catalyst [1].

The oxygen sensor detects oxygen by slowly reacting with O_2_, producing a current, and lasts for ∼ 2 years under ambient oxygen levels. In order to maximize the sensor lifetime, it should be stored in an anaerobic environment, if the anaerobic chamber is not in use.

## Validation and characterization

**Fig. 10:**
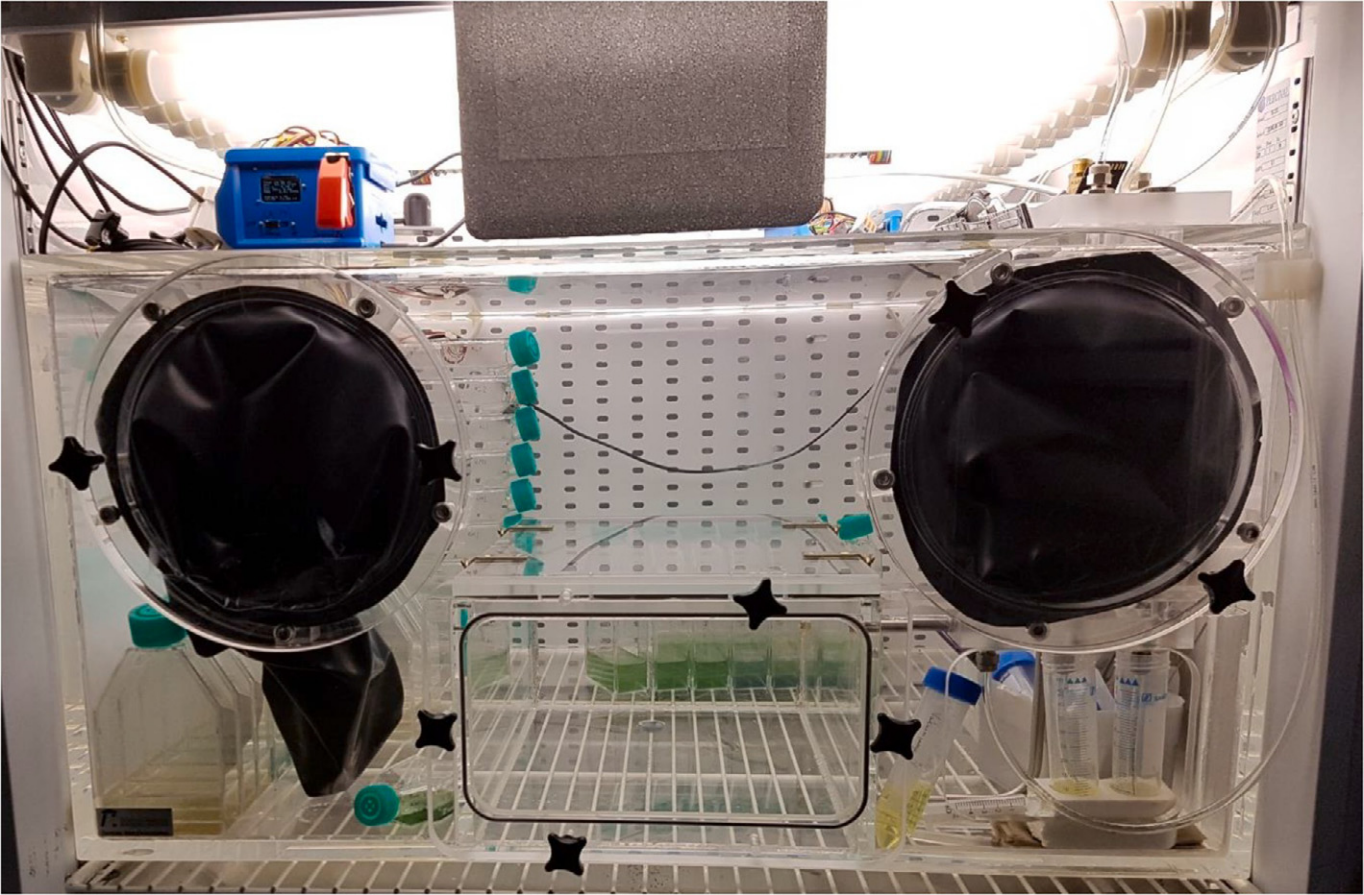
Anaerobic chamber fully functional and wired inside a culture chamber with growing cyanobacterial cultures in ventilated culture flasks.

The anaerobic chamber described in this paper was successfully used in studying the effects of Fe(II) on cyanobacteria. To do so, the anaerobic workstation was inserted inside a Percival culture chamber (E-22L), which controlled lighting, temperature and CO_2_ levels via an external sensor (Fig. 10). In order to test for the effect of photooxidation of Fe(II), or the buildup of oxygen inside the anaerobic chamber, control cultures without cyanobacteria were set up and Fe(II) measured throughout the whole experiment by means of the colorimetric ferrozine assay. The whole experiment was then repeated inside discrete, hermetically sealed, anaerobic bottles (which were also set up inside the anaerobic chamber) to test for the effects of O_2_ buildup on cyanobacterial growth.

**Fig. 11:**
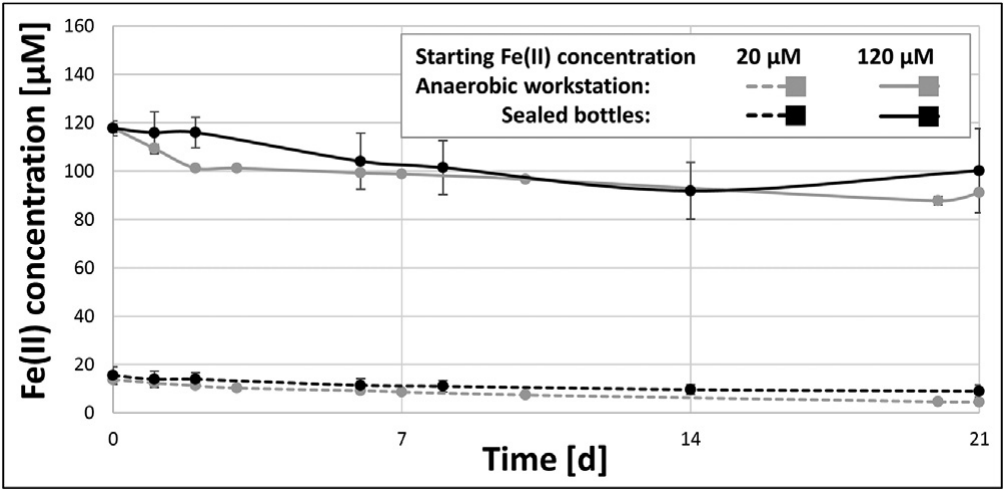
The Fe(II) concentrations of growth media over the course of 21 days, if either incubated inside the here described anaerobic chamber (grey), or in sealed anaerobic bottles (black). The starting Fe(II) concentrations were either set at 20 µM (dotted lines) or 120 µM (solid lines).

The Fe(II) measurements of the controls showed that the anaerobic box maintained an anerobic atmosphere throughout the whole experiment just as well as the individually sealed bottles (Fig. 11). Under aerobic conditions, the complete oxidation of Fe(II) would have been observed within 20 min. The slight drop in Fe(II) concentration over the whole length of the experiments is most likely the result of photo-oxidation, as the media was constantly exposed to the light needed for the growth of the cyanobacteria. This result clearly demonstrates the efficiency of the anaerobic workstation, as the control cultures in ventilated flasks were incubated at the same time as the cyanobacterial cultures inside the anaerobic workstation. Despite the cultures producing ∼ 170 ml of pure O_2_ per day near the end of the experiment, the Fe(II) in the control flasks was not oxidized. Without the automated injection of H_2_ and the regulation of humidity, this high influx of oxygen would have required manual user intervention at least every second day. An open bottle placed inside the anaerobic chamber during the experiments with media containing resazurin (20 mg × l^−1^), a commonly used indicator for anaerobic conditions, also showed no signs of oxygen in measurable quantities.

The anaerobic box was also used for the study of phosphorylation of redox sensitive proteins using radioactive ^32^P, hence the radioactive stickers in Fig. 4. The thick PMMA body of the anaerobic chamber offers better protection against beta-radiation then the thin plastic used in anaerobic tents, while the compact interior also allows for an easier clean-up after the end of the labeling experiments.

## Declaration of Competing Interest

The authors declare that they have no known competing financial interests or personal relationships that could have appeared to influence the work reported in this paper.
